# Interaction between a fluoroquinolone derivative KG022 and RNAs: Effect of base pairs 3′ adjacent to the bulged residues

**DOI:** 10.3389/fmolb.2023.1145528

**Published:** 2023-03-14

**Authors:** Rika Ichijo, Takashi Kamimura, Gota Kawai

**Affiliations:** ^1^ Graduate School of Engineering, Chiba Institute of Technology, Chiba, Japan; ^2^ Veritas In Silico Inc., Tokyo, Japan

**Keywords:** fluoroquinolone, RNA, interaction, NMR, specificity

## Abstract

RNA-targeted small molecules are a promising modality in drug discovery. Recently, we found that a fluoroquinolone derivative, KG022, can bind to RNAs with bulged C or G. To clarify the RNA specificity of KG022, we analyzed the effect of the base pair located at the 3′side of the bulged residue. It was found that KG022 prefers G-C and A-U base pairs at the 3′side. Solution structures of the complexes of KG022 with the four RNA molecules with bulged C or G and G-C or A-U base pairs at the 3′side of the bulged residue were determined to find that the fluoroquinolone moiety is located between two purine bases, and this may be the mechanism of the specificity. This work provides an important example of the specificity of RNA-targeted small molecules.

## 1 Introduction

RNA-targeted small molecules are recognized as a promising modality in drug discovery to be developed (Warner et al., 2018; Giorgio et al., 2019; Sztuba-Solinska et al., 2019). Some natural antibiotics, including aminoglycosides, tetracyclines and macrolides, are known to bind to ribosomal RNAs for their antiviral activities (Aboul-ela, 2006). Many kinds of drug-like small compounds such as ribocil (Howe et al., 2015) and branaplam (Palacino et al., 2015) were found to bind to RNAs in structure-specific manner. Furthermore, nucleobase specific compounds were developed, and it was demonstrated that the naphthyridine carbamate dimer (NCD), which binds specifically to guanine bases, alleviates disease phenotype in *Drosophila* model by binding RNAs with UGGAA repeats (Shibata et al., 2021). As the first RNA-targeted small molecule drug, risdiplam has been approved by US Food and Drug Administration as a treatment for spinal muscular atrophy (Ratni et al., 2021). Risdiplam is known to promote the exon seven inclusion in the human survival of motor neuron 2 (SMN2) transcript (Ratni et al., 2021) by stabilizing the interaction between the 5′splicing site and U1 snRNA. Although branaplam as well as risdiplam bind to the RNA and U1-C complexes, binding sites of these compounds are thought to be the RNA component (Palacino et al., 2015; Sivaramakrishnan et al., 2017; Tang et al., 2021). Thus, it is important to enlarge the chemical space for the RNA binding small molecules.

**FIGURE 1:**
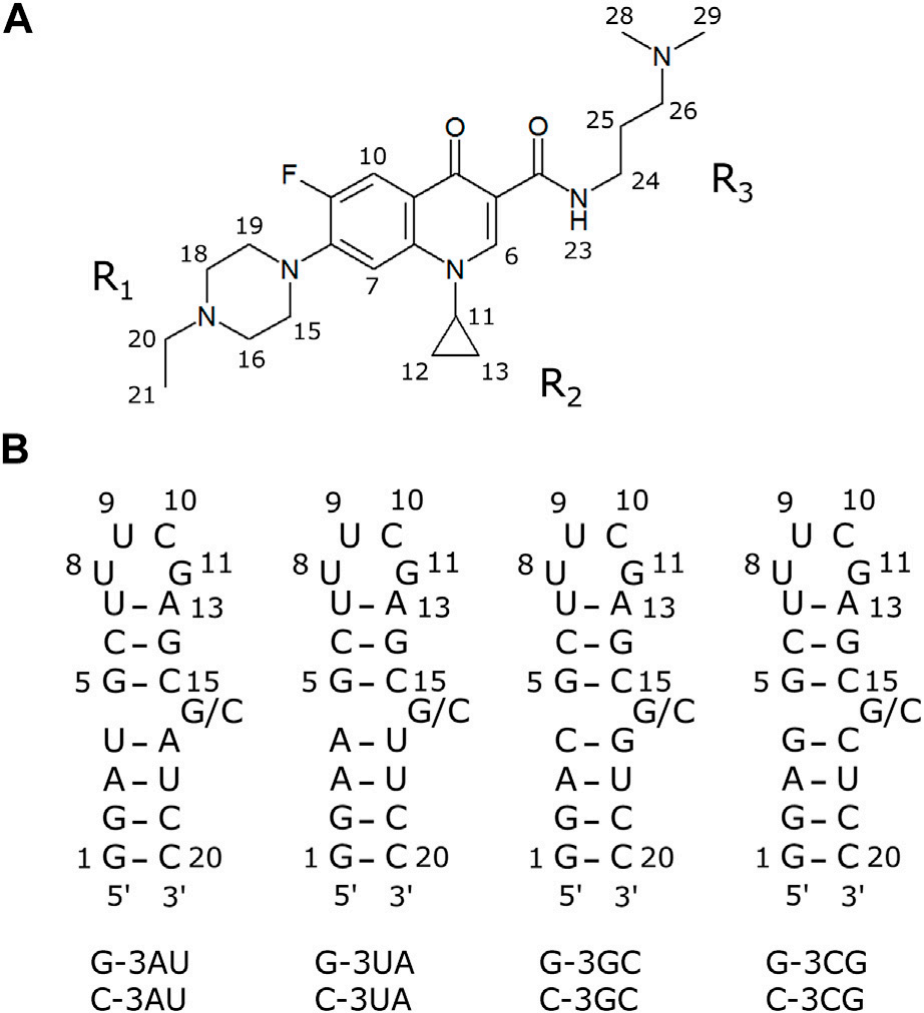
The compound and RNAs used in this study **(A)** A fluoroquinolone derivative KG022. **(B)** Model RNAs used in this study.

Recently, we have demonstrated that a fluoroquinolone derivative, KG022 (Figure 1A), shows specific binding to the region with the single bulged C or G of hairpin RNAs (Nagano et al., 2022). KG022 binds between the two base pairs of the bulge region and the cyclopropane ring of KG022 and the bulged base are close to each other. KG022 is similar to ciprofloxacin (CPFX) which is one of the most successful and widely used fluoroquinolone drugs for the treatment of a wide range of infectious diseases (Sharma et al., 2009). CPFX has a piperazine group in R_1_, a cyclopropane ring in R_2_ and a carboxyl group in R_3_. CPFX and its relating compounds have enhanced pharmacokinetic properties as well as extensive and potent activities against various parasites, bacteria and mycobacteria (Sharma et al., 2009). Thus, fluoroquinolone derivatives can be promising candidates for RNA-targeted drugs.

To further characterize the binding specificity of KG022 to RNAs, model RNAs with various base pairs at the 3′side of the bulged residue were prepared (Figure 1B) and their interactions with KG022 were analyzed by NMR spectroscopy. It was demonstrated that KG022 prefers GC and AU base pairs rather than CG and UA base pairs at the 3′side of the bulged residue. The basis of the specificity was discussed based on the solution structures of the RNA-KG022 complexes.

## 2 Materials and methods

### 2.1 Design of RNAs and sample preparation

Hereafter the four RNAs with 19 residues were called, for example, as G-3AU indicating an RNA with A residue at the 3′side of the bulged G (Figure 1B). It is noted that the loop sequences are different from RNAs used for previous work. Notably, for both cases, KG022 did not affect the NMR signals of the loop. The same residue numbers with the previous work were used as shown in Figure 1B.

RNA samples were purchased from Hokkaido System Sciences Co., Ltd. The RNA samples were dissolved in 20 mM sodium phosphate buffer (pH 6.5) with 50 mM sodium chloride and 5% D_2_O. KG022 sample was provided by Taiyo-Nippon Sanso Corporation. KG022 sample was dissolved to be 10 mM in water. For NMR experiments, RNA concentrations were 0.22–0.45 mM. The G-3GC with 10% [^13^C/^15^N]G at the residue 17 was purchased from Taiyo-Nippon Sanso Corporation to obtain a 0.25 mM solution for NMR measurements.

### 2.2 NMR measurements and analysis

All NMR spectra were measured with the AvanceNeo 600 spectrometer (Bruker Biospin) at 288 K. For the measurements of imino proton spectra, the water signal was suppressed by a jump-and-return pulse (Plateau and Guéron, 1982). For other spectra, the water signal was suppressed by a 3-9-19 pulse (Piotto et al., 1992). NMR spectra were processed with TopSpin (Bruker Biospin) and analyzed with Sparky (Goddard and Kneller, 2008). Titrations of RNAs by KG022 were performed by the addition of small amount of 10 mM KG022 solution up to the molar ratio 1.5.

### 2.3 Structure determination

Structures of the complexes were calculated with CNS_SOLVE (Brünger et al., 1998). Restraints used for the structure calculations were summarized in Table 1. In general, structure determination was done by the same method with the previous study (Nagano et al., 2022). For G-3AU and G-3GC, the glycosidic bond of G16 was fixed to the *syn*-conformation. For C-3AU and C-3GC, the sugar pucker of C16 was fixed to the C2′-*endo* form and the conformation of the glycosidic bond of C16 was fixed to the *anti*-conformation. For the four RNAs, the sugar packer of C20 was not fixed. Because imino proton signals for the five base pairs except for the terminal base pairs, G1-C20 and U7-A13, were observed, formation of RNA-A helix for the stem region was confirmed for each complex. The backbone structures for C15-G/C16–A/G17 and U/C4–G5 were not fixed. For the loop region, constraints of the C3′-*endo* form for U8 and G12, the C2′-*endo* form for U9 and C19, the *anti*-conformation for U8–C10, and the *syn*-conformation for G12 were used. Hydrogen bonds for O2 of U8 and H1 of G11 as well as HO2′ of U8 and O6 of G11 were also assumed. Structure calculations were performed 100 times to obtain 70, 48, 82 and 54 accepted structures for G-3AU, G-3GC, C-3AU and C-3GC, respectively. For C-3AU and C-3GC, the orientation of the cyclopropane moiety, C2-N1-C11-C12, was fixed to 120.0 ± 30.0. For C-3AU, the lower boundary for the NOE distance restraints for intermolecular NOEs in the weak range were increased by 0.5 Å. For C-3GC, the lower boundary for the NOE distance restraints for intermolecular NOEs in the weak range were increased by 1.0 Å and in the medium and strong ranges by 0.5 Å. For C-3AU, force constants for the dihedral angle restraints were increased from 1.0 to 2.0. NOEs for methyl and some methylene protons of KG022 are treated by the r^−6^ average mode. Stereo specific assignments were made for some protons of the cyclopropane and piperazine ring. Chemical shifts of assigned protons were shown in Supplementary Tables S1–S5. Determined structures were submitted with the chemical shifts and restraints to the protein data bank as the accession numbers of 8I46, 8I45, 8I44 and 8I43 for G-3AU, G-3GC, C-3AU and C-3GC, respectively. Structural statistics were shown in Table 1. Molecular images were prepared with UCSF Chimera (Pettersen et al., 2004).

**TABLE 1:** NMR restraints and statistics.

	RNA-G-3AU	RNA-G-3GC	RNA-C-3AU	RNA-C-3GC
Number of experimental restraints				
Distance restraints^a^	197	208	172	194
Intra-residue (RNA)	38	34	36	38
Sequential (RNA)	41	37	25	27
Medium range (RNA)	12	13	5	9
Long range (RNA)	23	33	33	23
Inter-molecule^b^	13	31	13	27
Intra-KG022^b^	50	39	40	49
Hydrogen bonding	20	21	20	21
Dihedral restraints	159	159	160	160
Planarity for base pairs	8	8	8	8
Heavy-atoms r.m.s. deviation (Å)^c^				
All	0.438 ± 0.217	1.270 ± 0.519	1.145 ± 0.460	0.554 ± 0.211
All (pairwise)	0.507 ± 0.132	1.226 ± 0.364	1.348 ± 0.351	0.598 ± 0.154
Backbone	0.422 ± 0.243	1.278 ± 0.515	1.200 ± 0.497	0.585 ± 0.219
Backbone (pairwise)	0.502 ± 0.178	1.161 ± 0.424	1.413 ± 0.398	0.567 ± 0.193
Stem (1–7, 13–15, 16–20)	0.414 ± 0.215	0.694 ± 0.288	1.008 ± 0.433	0.386 ± 0.185
Stem (pairwise)	0.502 ± 0.136	0.706 ± 0.185	1.124 ± 0.464	0.444 ± 0.162
R.m.s.d. around the ideal values				
bonds (Å)	0.0041 ± 0.00003	0.0046 ± 0.00003	0.0038 ± 0.00005	0.0066 ± 0.00002
angle (°)	1.0813 ± 0.0076	1.2476 ± 0.0224	1.0108 ± 0.0117	1.4322 ± 0.0020

^a^ The NOESY, spectra obtained with the mixing time of 200 ms were used. Stereo specific assignments were assumed for the methylene protons observed individually.

^b^ Each NOE, for the overlapped methyl or methylene protons was count as one.

^c^ Averaged r.m.s.d. between an average structure and the 10 converged structures were calculated. The converged structures did not contain experimental distance violation of >0.5 Å or dihedral violation >5°.

## 3 Results

### 3.1 Structure analysis of model RNAs without ligands

**FIGURE 2:**
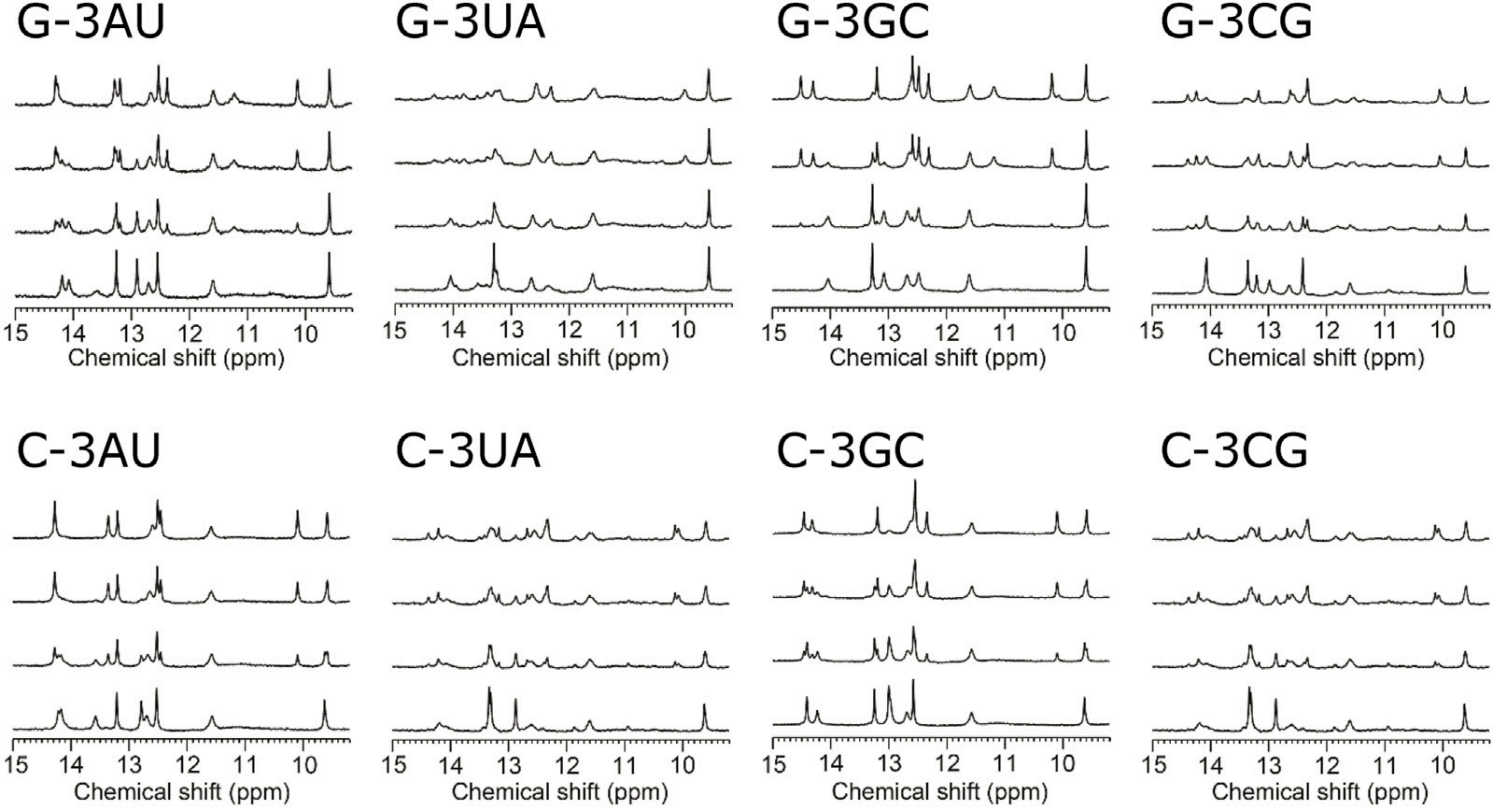
Titration of model RNAs by KG022 monitored by imino proton signals Molar ratio s of RNA and KG022 were 1:0, 1:0.5, 1:1 and 1:1.5 from bottom to top, respectively. ^1^H-NMR spectra measured with the jump-and-return pulse were shown.

To confirm the conformation of the RNAs shown in Figure 1B, NMR spectra of the RNAs were analyzed in free forms. As shown in the bottom spectra for each panel in Figure 2, signals for the UUCG loop were observed at 11.6 and 9.6 ppm for all four RNAs, indicating the formation of the loop structures. For all four RNAs, sharp imino proton signals of G14 were observed around 13.3 ppm, suggesting that the neighboring G5-C15 base pair is formed although the imino proton signal of G5 and amino proton signals of C15 were not observed due to exchange between these protons and water protons.

For G-3UA and G-3CG, inter-residual NOEs between C15H1′ and U17H6/C17H6 were observed, suggesting that C15 and U17/C17 are stacked to each other and G16 is flipped out. No inter-residual NOEs were observed for G16H8 of G-3UA and the signal for G16H8 for G-3CG was overlapped to other protons and NOEs could not be analyzed. In contract, for G-3AU, the inter-residual NOE between C15H1′ and A17H6 was not observed. Instead, inter-residual NOEs between C15H1′ and G16H8 as well as G16H1′ and A17H8 were observed, suggesting that G16 is located between C15 and A17. In the case of G-3GC, the signal of G16H8 was not observed due to the broadening. Based on the existence of amino proton signals and their chemical shifts, C4 and C15 were found to form G-C base pairs, probably with G17 and G5, respectively, and G16 is located between C15 and G17 with structural fluctuations. Notably, signals of C4H6 and U18H5 were broadened, and signals of C15H6 and G17H8 were slightly broadened, suggesting the conformational fluctuation around the bulge region. The assignment of the G17H8 was confirmed by using the residue-specifically ^13^C/^15^N-labeled G-3GC RNA (Supplementary Figure S1) and the sequential NOE connectivity between G17 and U18 was confirmed.

For RNAs with bulged C, the H5 and H6 of C15 resonate higher field (5.41–5.45 and 7.59–7.66 ppm) and those of C16 resonate lower field (5.56–5.82 and 7.79–7.96 ppm), suggesting that C15 is located in the stem and C16 is flipped out. For C-3UA, no inter-residual NOEs from C16H6 were observed. For C-3CG, based on the existence of amino proton signals and their chemical shifts, C17 was confirmed to form a G-C base pair. No inter-residual NOEs were observed for C16H6, consistent with that C16 is flipped out. For C-3AU, the inter-residual NOE between C16H1′ and A17H8 was observed, whereas the inter-residual NOE between C15H1′ and C16H6 was not resolved due to the signal overlap. For C-3GC, a weak inter-residual NOE between C15H1′ and G17H8 was observed. The signal of C15H6 was slightly broadened and the signal of C16H1′ could not assigned probably due to the signal overlap.

Although the conformations of C16 and G16 are varied to each other, all model RNAs were confirmed to form the secondary structures shown in Figure 1B. It is noted that solution structures of the four RNAs in free form were determined tentatively (Supplementary Figure S2).

### 3.2 Interaction of KG022 with model RNAs

Figure 2 shows the imino proton spectra during the titration by KG022. For all RNAs, the spectra were changed upon addition of KG022. In the case of G-3AU, some signals were decreased, and some new sharp signals appeared at the molar ratio 1:0.5, and the decreased signals were almost disappeared at the molar ratio 1:1. Only new sets of sharp signals for the RNA-KG022 complex were observed at the molar ratio 1.5, suggesting that KG022 binds to the RNA with molar ratio 1 for G-3AU. Sharp signals for the complex with KG022 were also observed for G-3GC, indicating the formation of stable complexes for G-3AU and G-3GC (Figure 2). In contrast, in the case of G-3UA and G-3CG, signals for the complex are rather broad, suggesting that the interaction is weak. Thus, KG022 shows base pair preferences. Similarly, sharp signals for the complex were observed for C-3AU and C-3GC. In contrast, for C-3UA and C-3CG, signals for the complex were broadened. Thus, it was found that A-U and G-C base pairs at the 3′side were preferred for RNAs with bulged G as well as bulged C.

**FIGURE 3:**
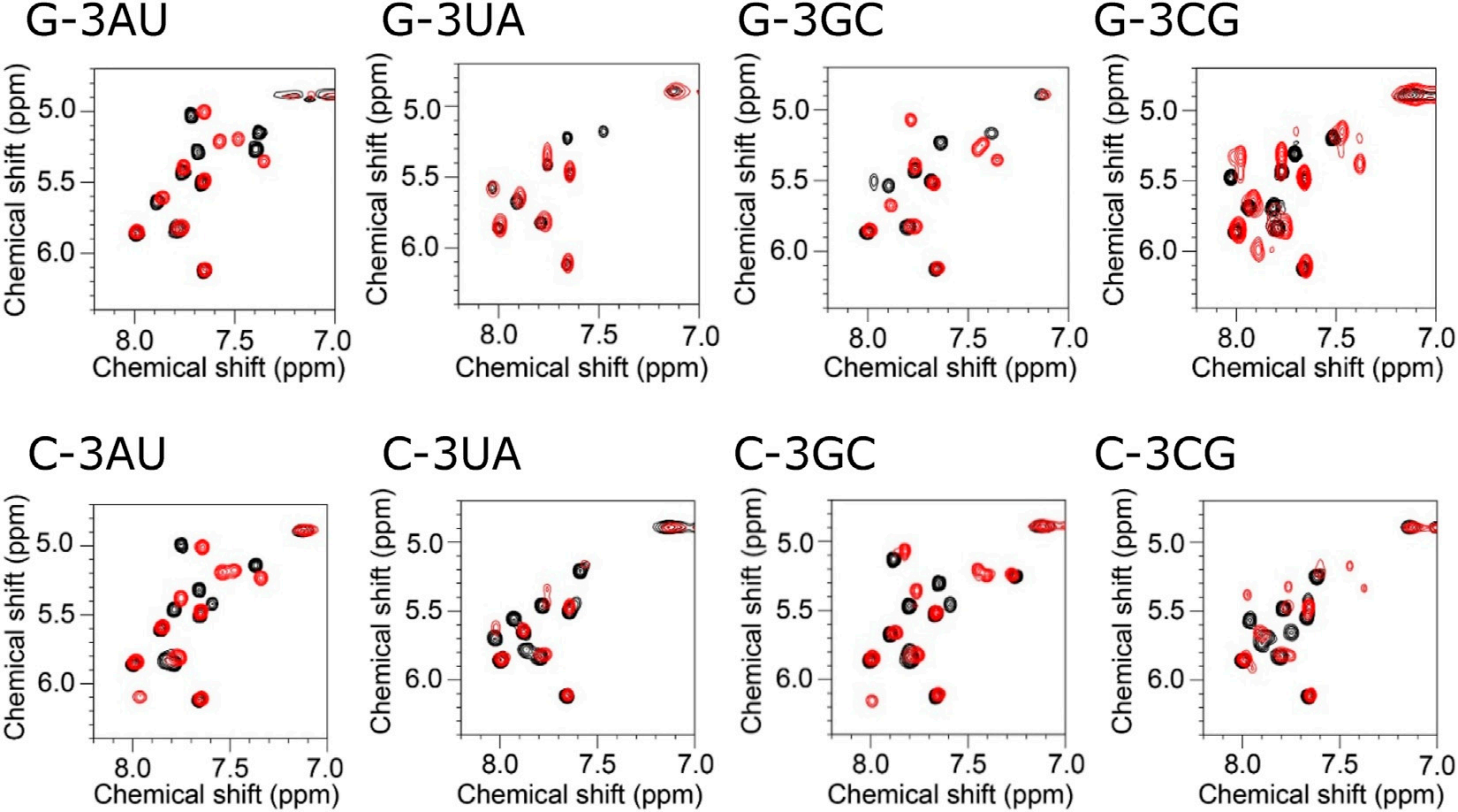
Titration of model RNAs by KG022 monitored by pyridine H5-H6 signals Molar ratio s of RNA and KG022 were 1:0 (black) and 1:1 (red). ^1^H-^1^H HOHAHA spectra measured with the 3-9-19 pules were shown.

Similar tendency was also observed for the H5-H6 signals in the HOHAHA spectra (Figure 3). For G-3AU and G-3GC, some specific signals were shifted upon addition of KG022. In contrast, for G-3UA, some signals were disappeared probably due to broadening. For G-3CG, number of signals was increased, suggesting the structural polymorphism. These results indicated that the A-U and G-C base pairs at the 3′side were preferred for RNAs with bulged G. Similarly, some specific signals were shifted for C-3AU and C-3GC upon addition of KG022. Again, some signals were disappeared for C-3UA and number of signals were increased for C-3CG. Thus, it was confirmed that A-U and G-C base pairs at the 3′side are preferred for RNAs with bulged G and C.

**FIGURE 4:**
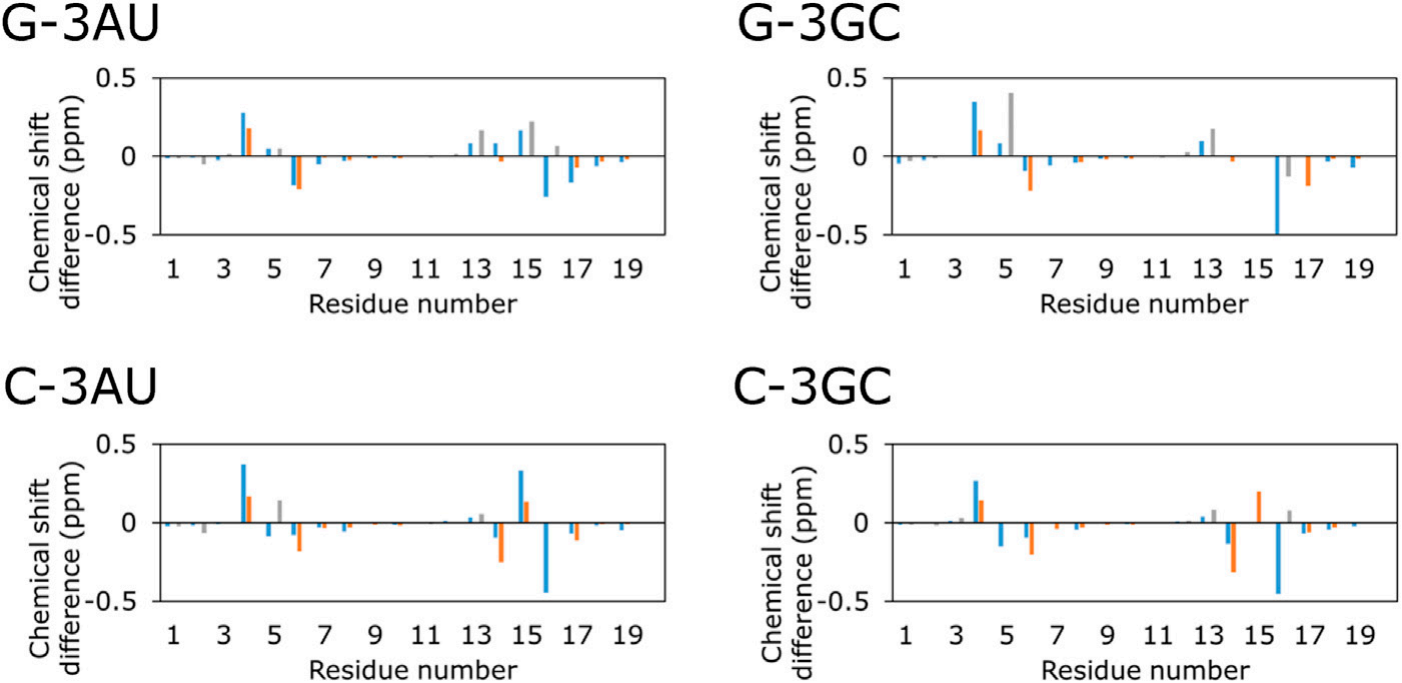
Chemical shift changes upon addition of KG022 Blue: H1′, Orange: H6, Gray: H8.

Then, NOESY spectra were analyzed for the RNAs which form the stable complex with KG022, G-3AU, G-3GC, C-3AU and C-3GC. For all RNAs, most of signals for H8/H6/H2 and H1′/H5 were assigned in the complex forms (Supplementary Figure S3 and Supplementary Tables S1–S4). Figure 4 shows the chemical shift differences between the free and complex forms. For all four RNAs, the 4th residue showed down-field shift. C6 and the 16th residue showed up-field shift. For RNAs with bulged C, G14 showed up-field shift whereas, for RNAs with bulged G, A13 showed down-field shift. In the case of G-3GC, G5H8 showed down-field shift. For all RNAs, residues with larger chemical shift changes were located around the bulged residues, indicating that KG022 binds to the region. It is noted that the imino proton signal for G5 and amino proton signals for C15 were observed in the complexes with KG022, and those were not observed in the free form. Thus, the G5-C15 base pair was stabilized upon binding of KG022.

**FIGURE 5:**
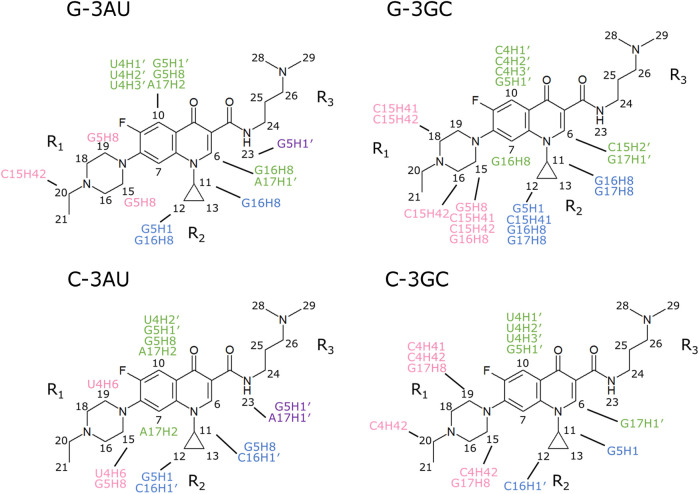
Intermolecular NOEs observed for the four complexes NOESY spectra obtained with the mixing time of 300 ms were used. Intermolecular NOEs with fluoroquinolone ring, R_1_, R_2_ and R_3_ were indicated by green, pink, blue and purple, respectively.

Inter-molecular NOEs were analyzed by using the NOESY spectra obtained with the mixing time of 300 ms (Figure 5). The number of inter-molecular NOEs identified were 15, 20, 14 and 13 for G-3AU, G-3GC, C-3AU, and C-3GC, respectively. Notably, each NOE for the overlapped methyl or methylene protons was count as one. The inter-molecular NOEs observed for all four RNAs were G5H1′-K21H10 and U/C4H2′-K21H10, indicating that H10 of KG022 is located opposite to the bulged residues. G16H8-K21H11 was shared with G-bulge RNAs and C16H1′-K21H122 was shared with C-bulge RNAs, indicating that the relative position of the cyclopropane ring to the bulged residue is slightly different between RNAs with bulged G and C. The number of inter-molecular NOEs for RNAs with bulged G are larger than bulged C, suggesting that stability of the complex is higher for RNAs with bulged G than bulged C.

### 3.3 Solution structures of RNA-KG022 complexes

As described above, NMR signals of the four complexes were assigned by the conventional method. For G-3GC, G17 was labeled by 10% ^13^C/^15^N to confirm the assignments of G16 and G17 (Supplementary Figure S1). Intermolecular NOEs observed with the mixing time of 200 ms were used for the structure calculations.

**FIGURE 6:**
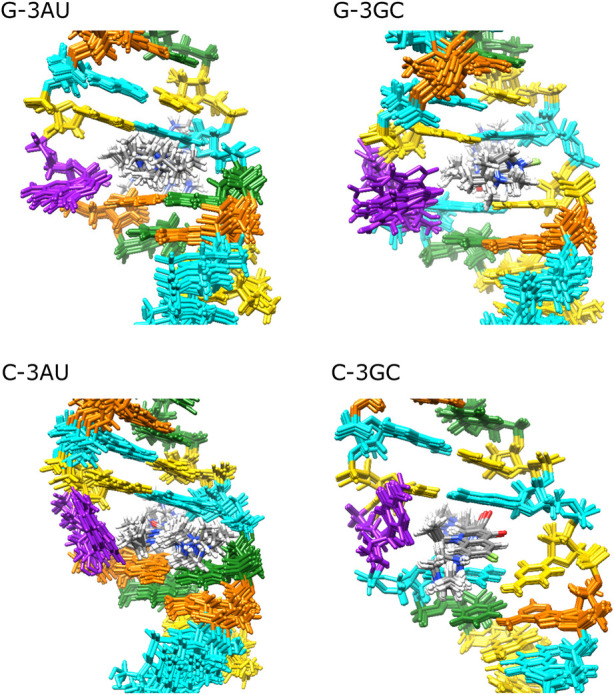
Solution structures of complexes between RNAs and KG022 Views from the major groove were shown. For each RNA, 10 lowest energy structures were superposed. Purple: G16 or C16, Cyan: G, Orange: A, Yellow: C, Green: U.

**FIGURE 7:**
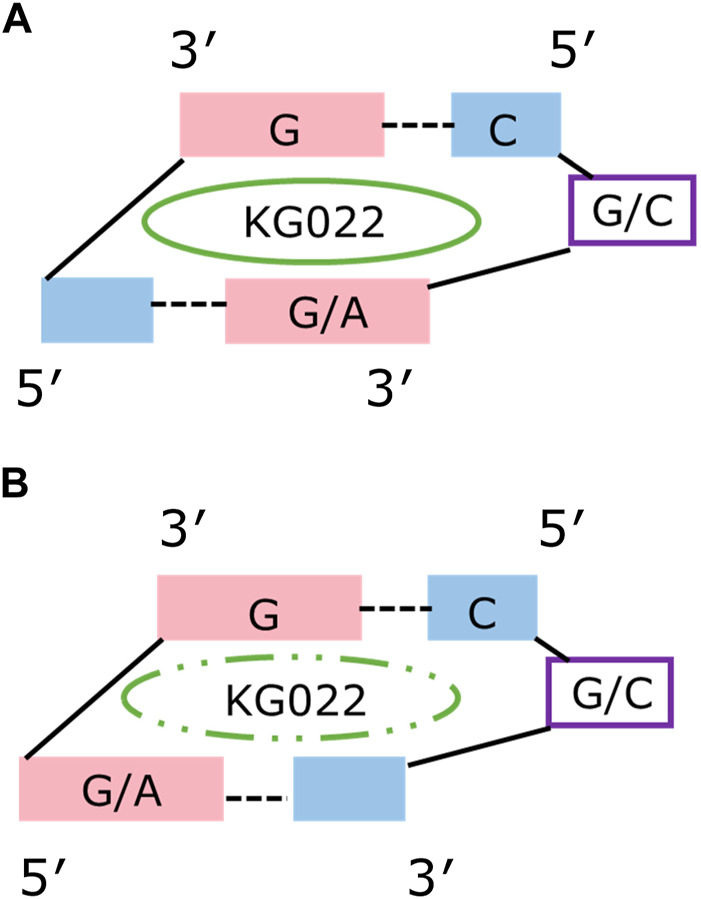
Schematic drawing of the KG022 binding site Views from the minor groove were shown. **(A)** RNAs with purine-pyrimidine base pairs at the 3’side of the bulged residues. **(B)** RNAs with pyrimidine-purine base pairs at the 3’side of the bulged residues.

Solution structure for G-3AU was determined as shown in Figure 6 to confirm the similar binding mode with the previous result (Nagano et al., 2022). Similar results were obtained for G-3GC, C-3AU and C-3GC. The shared characteristics for these complex structures are the interaction between the cyclopropane ring of KG022 and the bulged base, G16 or C16, and the continuous stacking among G5, the fluoroquinolone moiety of KG022 and the purine base, A17 or G17, as shown in Figure 7A. Thus, it was suggested that the origin of the base pair preference is caused by the locations of the three substituents to make the stable interactions of KG022 in the binding pockets of the model RNAs. Although the conformation of the bulged residues were not well defined, conformational differences were obvious; G16 is in the C3′-*endo*-*syn* conformation whereas C16 is in the C2′-*endo*-*anti* conformation. Notably, proton chemical shifts of the cyclopropane ring were different between RNAs with bulged G and C (Supplementary Figure S4).

## 4 Discussion

This study demonstrated how the specific binding of a small compound is achieved. KG022 prefers G-C and A-U base pairs to form stable stacking of G, fluoroquinolone and G/A. It is known that the self-stacking tendency of the various nucleobase residues decreases in the order adenine, guanine and cytosine/uracil (Sigel et al., 2014). Analysis of the effect of the base pairs at the 5′side of the bulge residue will give further information on the RNA-binding specificity of KG022. Although the RNA-specificity of KG022 was analyzed only with the five residues among the bulge regions in this experimental framework, precise evaluation as well as prediction of the specificity in the context of precise geometry can be done by computational methods based on the complex structures determined.

In the free forms of the model RNAs, the bulged residues were flipped out and flanking base-pairs were stacked to each other for G-3UA, G-3CG and RNAs with C bulge. It is possible that the closed structures in the bulged region for those RNAs in free form prevent the entry of KG022 to the binding sites. In contrast, for G-3AU and G-3GC, the bulged G residue is located between the neighboring base pairs which may provide space for KG022 binding. However, the broadening of the NMR signals for G/C-3UA and G/C-3CG indicating the conformational fluctuations. Notably, the bound KG022 was not eluted by the centrifugal ultrafiltration for G/C-3AU and G/C-3GC but partly eluted for G/C-3UA and G/C-3CG (Supplementary Figure S5), also suggesting the difference in the stability of the complexes. Single bulges were classified into the groups I, II and III (Blose et al., 2007; Mccann et al., 2011; Kent et al., 2014). G-3UA and G-3CG belong to the group I, where the position of the bulge is unambiguous with a bulged nucleotide that is not identical to either of the neighboring nucleotides. For the group II, the bulged nucleotide is identical to one of its nearest neighbors, and G-3GC and the four RNA with the bulged C belong to this group. The group III is the bulged nucleotide of either AG/U or CU/G, and G-3AU belongs to this group. It is possible that the conformational fluctuation for the groups II and III affects to the binding affinity of KG022.

KG022 consists of the fluoroquinolone moiety and three substituents as shown in Figure 1A. The cyclopropane group (R_2_) is located close to the bulged residue, suggesting that this moiety is important to accommodate in the bulged region. The ethyl piperazine group (R_1_) is located in the major groove. Although inter-molecular NOEs between the ethyl piperazine group and the bulged residues were found only for G-3GC, it is possible that this group is the determinant for the G and C specificity as the bulged residue. The dimethyl aminopropyl group (R_3_) is located in the minor groove. Probably, the tertiary ammonium residue interacts to the phosphate groups of the RNA backbone. By changing the combination of these substituents, the sequence and/or structural specificity of the fluoroquinolone derivatives may be altered. Thus, this work demonstrated that the fluoroquinolone derivatives can be lead scaffolds for RNA-targeted drug discovery.

## Data Availability

The original contributions presented in the study are included in the article/Supplementary Materials, further inquiries can be directed to the corresponding author.
