# Supplementary material for: Interaction between a fluoroquinolone derivative KG022 and RNAs: Effect of base pairs 3′ adjacent to the bulged residues
Source: Front Mol Biosci. 2023 Mar 14;10:1145528. doi: 10.3389/fmolb.2023.1145528 (PMC10043337; doi:10.3389/fmolb.2023.1145528)
Supplement: Supplementary file 1 [file DataSheet1.PDF]

Interaction between a fluoroquinolone derivative KG022 and RNAs: effect of base pairs 3' adjacent to the bulged residues

Rika Ichijo, Takashi Kamimura and Gota Kawai

Supplemental materials

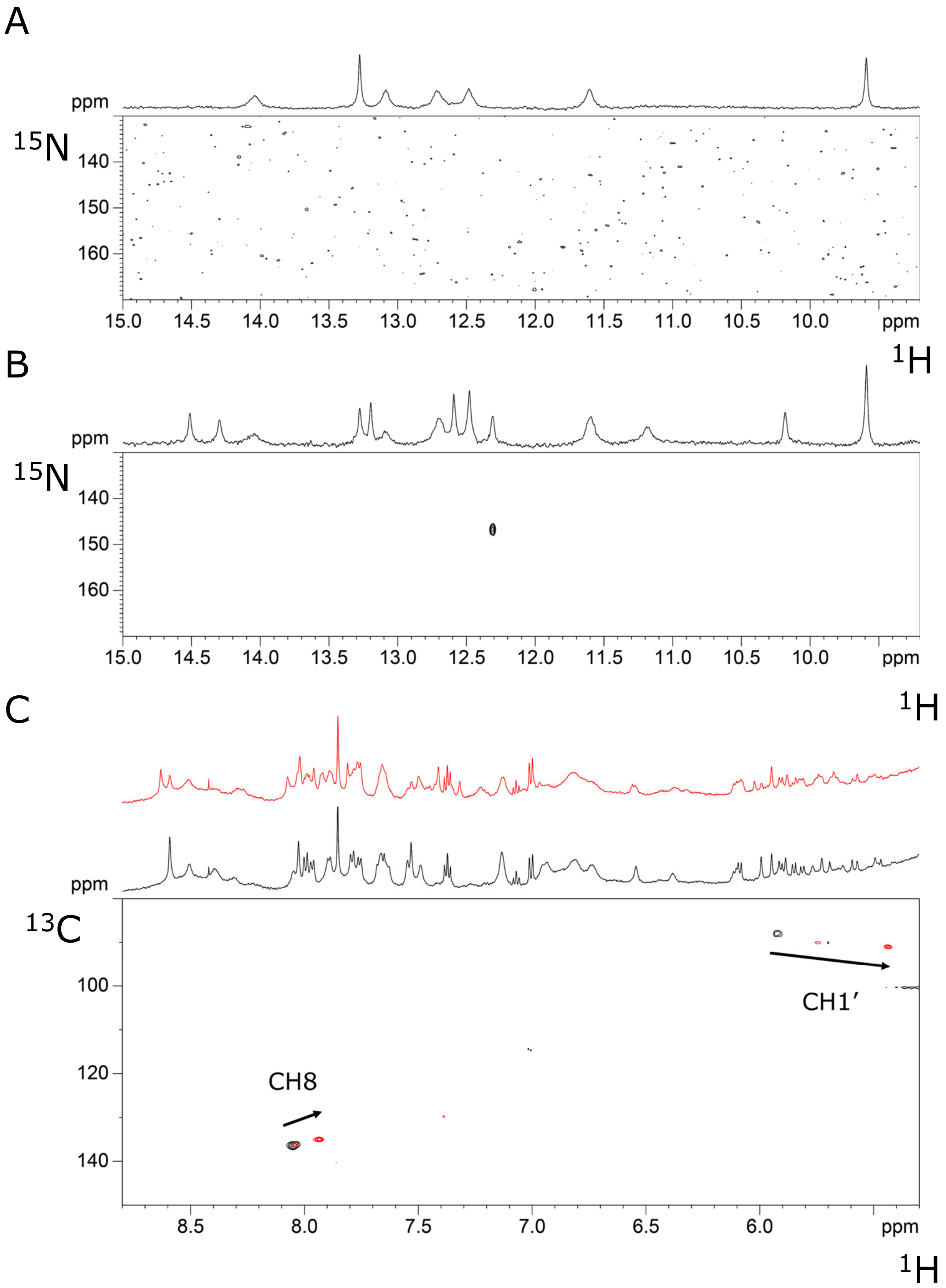

Supplemental Fig. S1. NMR spectra of [^{13}C/^{15}N]G17-labeled G-3GC
A, B: ^{15}N-^{1}H HMQC spectra in the absence and presence of KG022, respectively. C: ^{13}C-^{1}H SQC spectrum in the absence (black) and presence (red) of KG022.

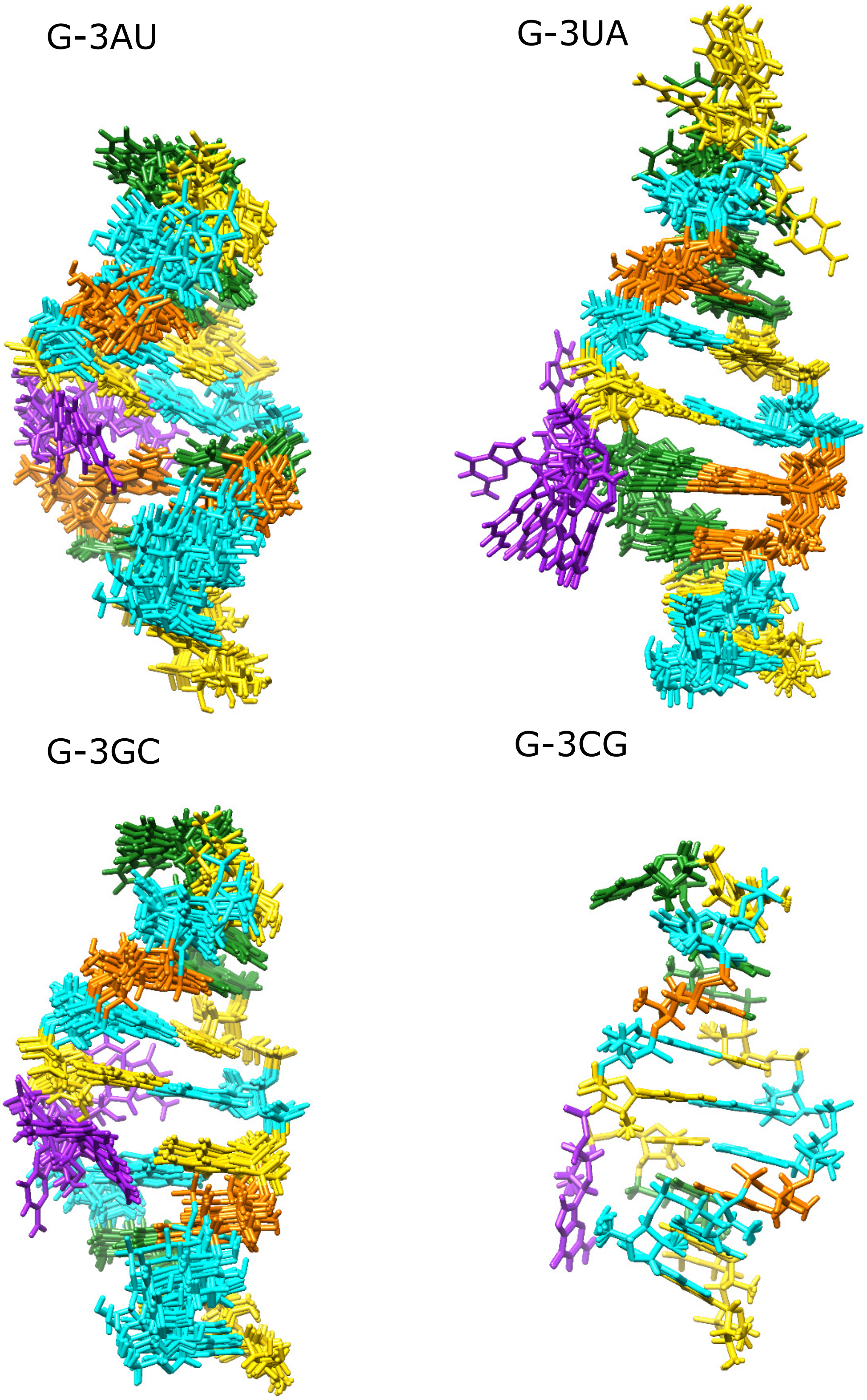

Supplemental Fig. S2. Tentative solution structures of the model RNAs in the free form

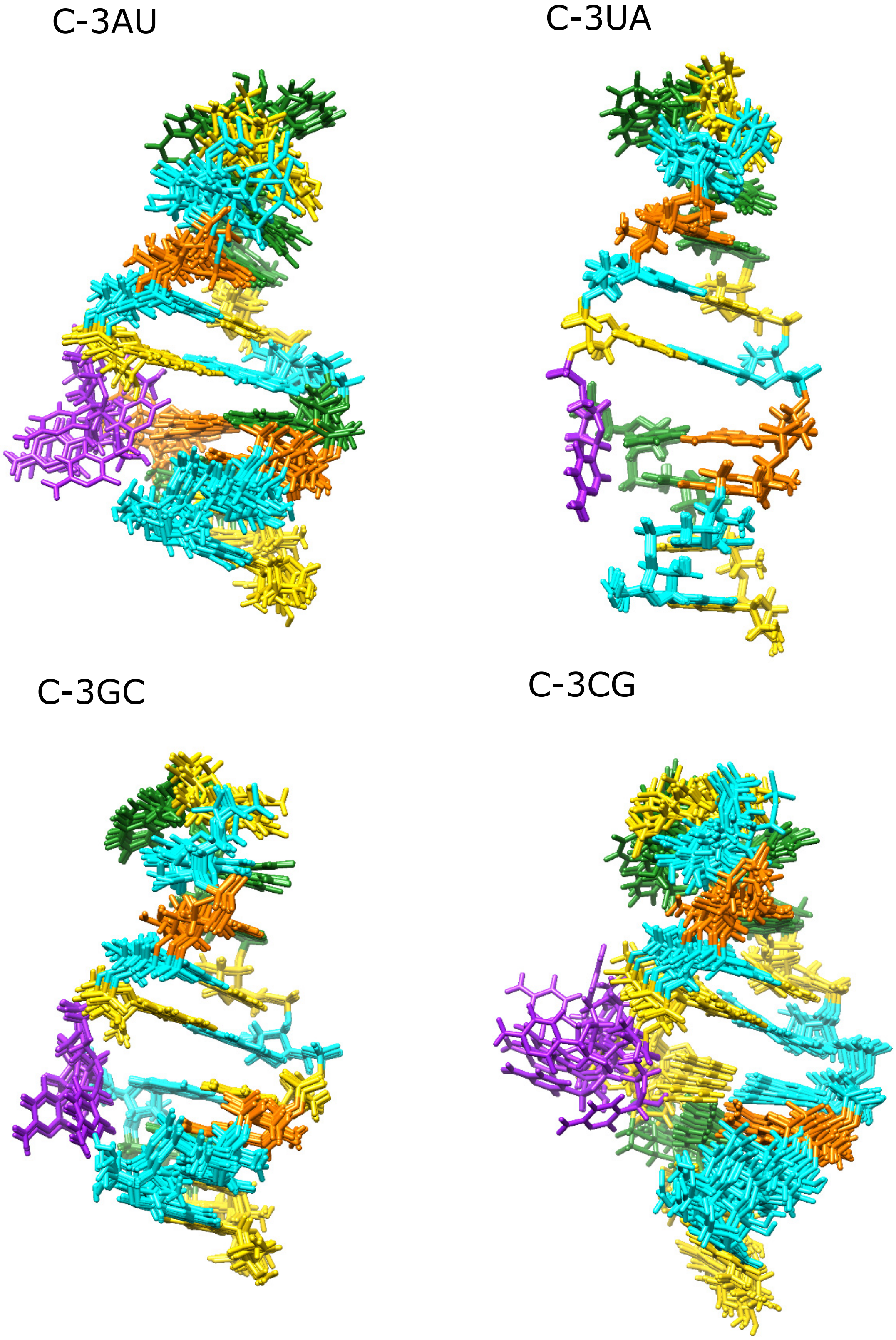

Supplemental Fig. S2. Continued

G-3AU

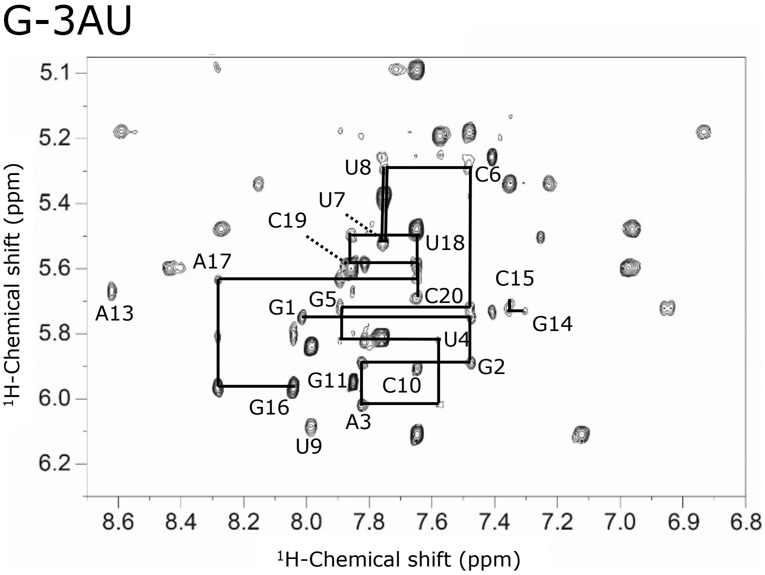

G-3GC

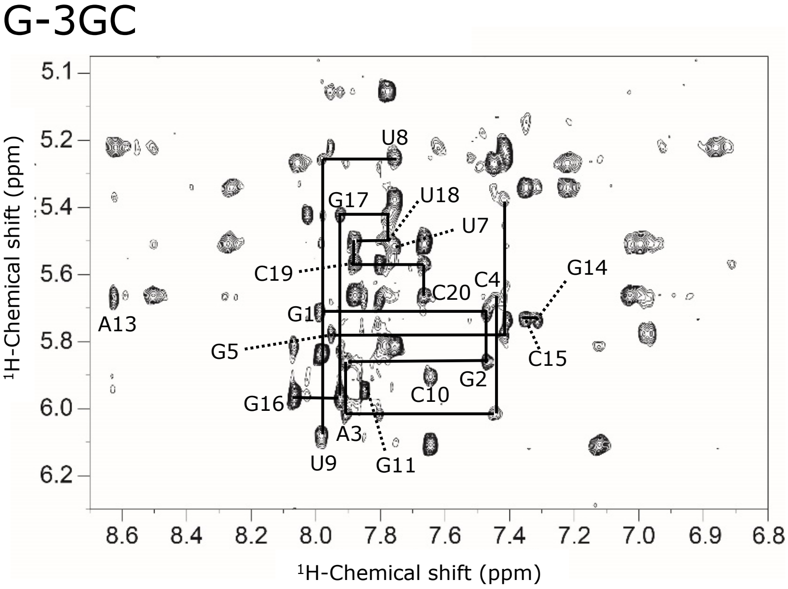

Supplemental Fig. S3. Sequential assignments of the four complexes

^{1}H-^{1}H NOESY spectra measured with the 3-9-19 pulse were shown. The mixing time was 200 ms.

C-3AU

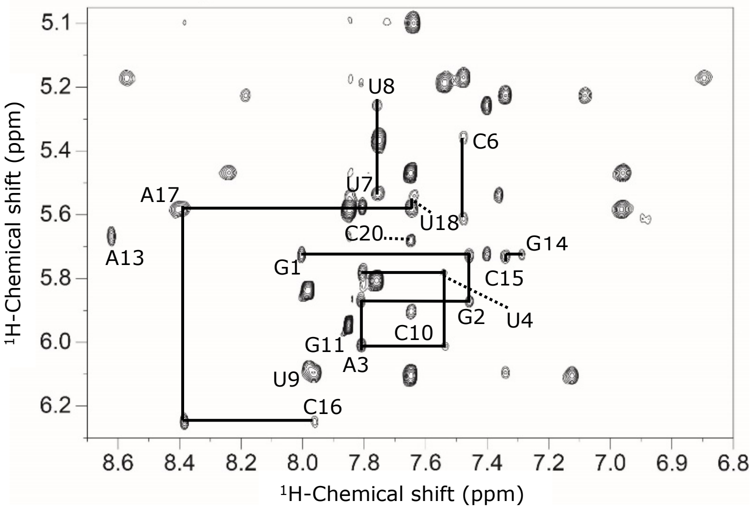

C-3GC

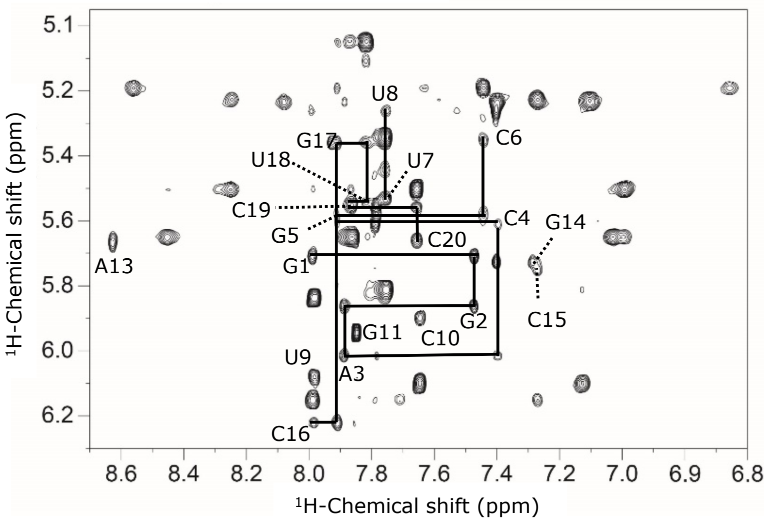

Supplemental Fig. S3. Continued

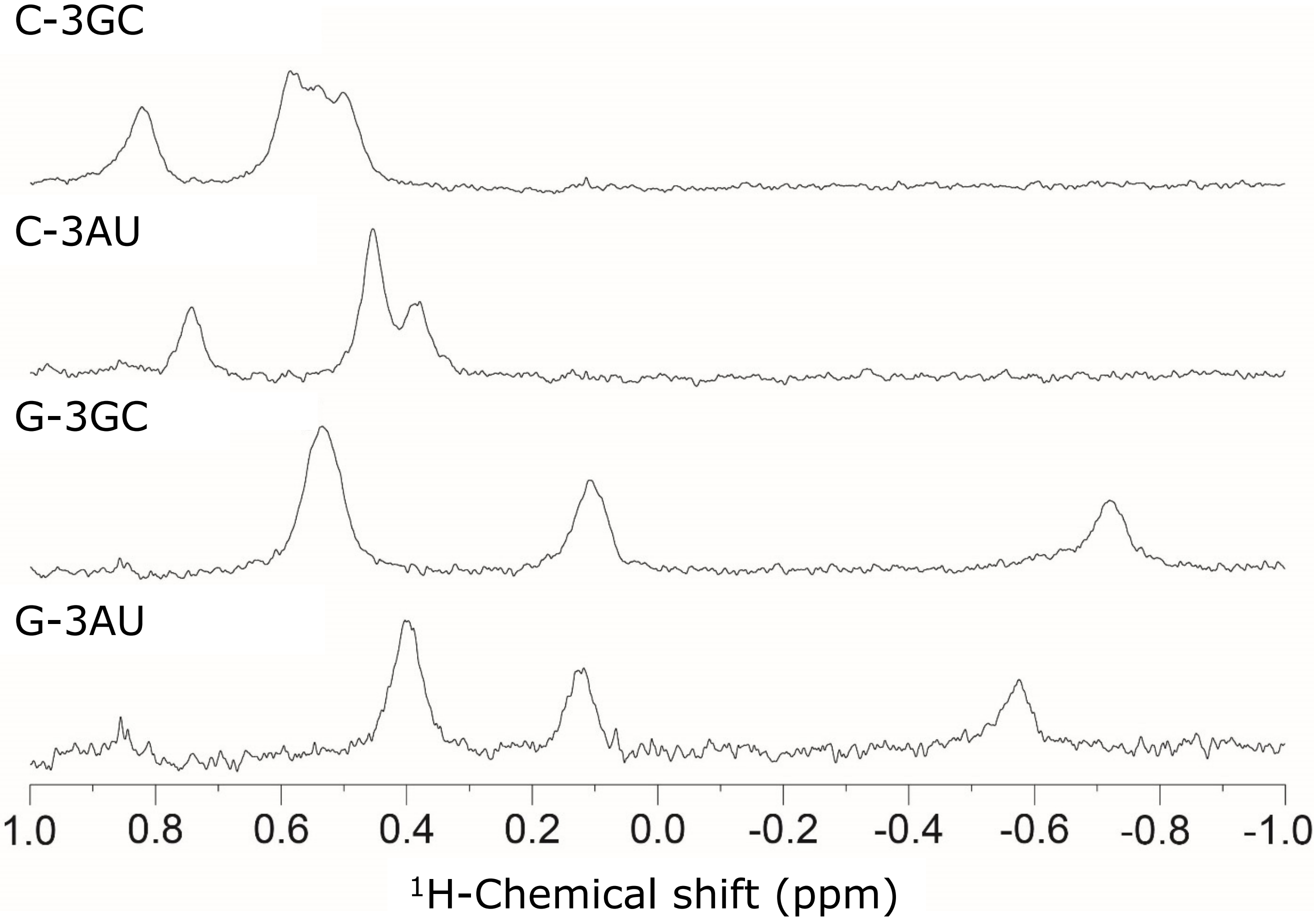

Supplemental Fig. S4. Signals of KG022 bound to target RNAs

Signals for the methylene protons of the cyclopropane ring are shown. ^{1}H-NMR spectra measured with the 3-9-19 pulse were shown.

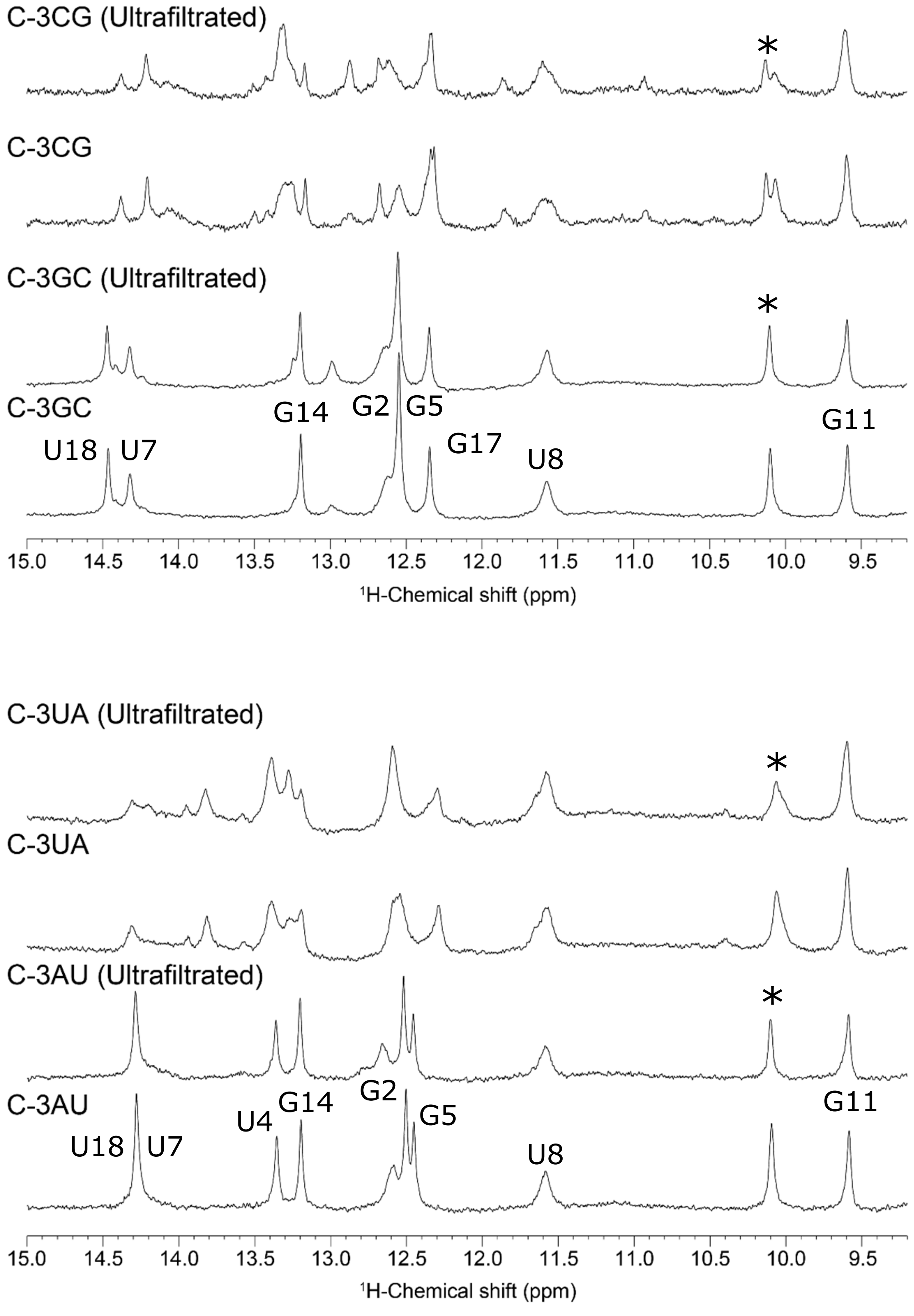

Supplemental Fig. S5. Imino proton spectra of RNAs before and after ultrafiltration

Signals for amido proton of KG022 are indicated by asterisks. The ultrafiltration was performed with vivaspin 2-3K (MWCO 3000, cytiva).

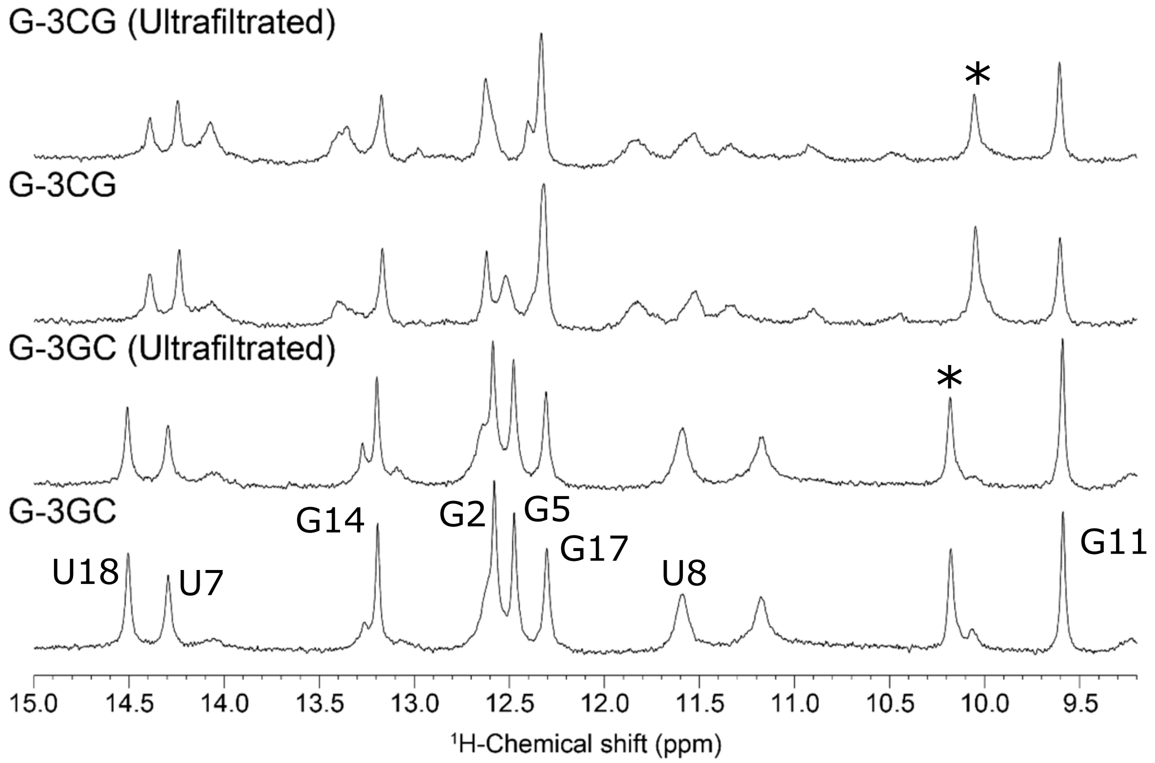

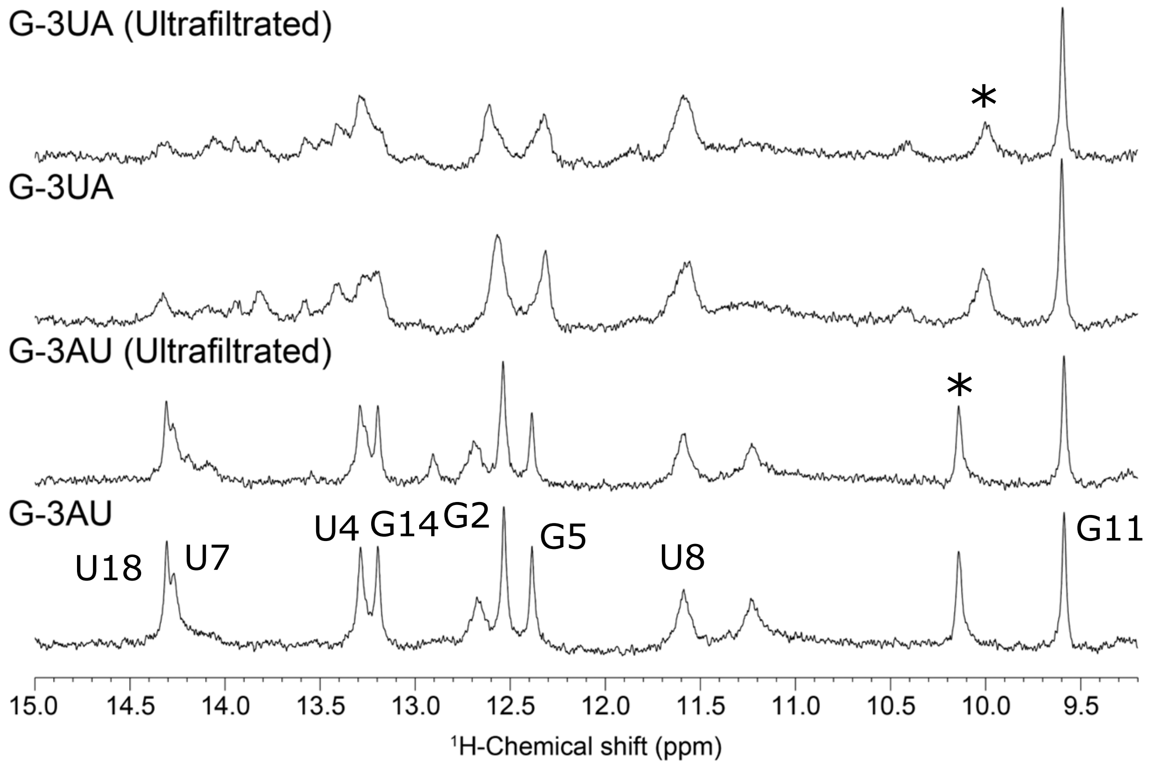

Supplemental Fig. S5. Continued
